# Lung Perfusion Imaging with Technetium-99m Macroaggregated Albumin should be Combined with Contrast-enhanced Echocardiography for the Diagnosis of Hepatopulmonary Syndrome

**DOI:** 10.4274/mirt.galenos.2020.52244

**Published:** 2020-10-19

**Authors:** Georgios Meristoudis, Georgia Keramida, Ioannis Ilias

**Affiliations:** 1 Hippokration General Hospital, Clinic of Nuclear Medicine, Thessaloniki, Greece; 2 Royal Brompton and Harefield, NHS Foundation Trust, Department of Nuclear Medicine, London, United Kingdom; 3 Elena Venizelou General Hospital, Clinic Endocrine Unit, Athens, Greece

**Keywords:** Hepatopulmonary syndrome, technetium-99m macroaggregated albumin, lung perfusion scintigraphy, right-to-left shunt, contrast-enhanced echocardiography

## Dear Editor,

We have read with great interest the recent article by Alipour et al. (1) regarding the diagnosis of hepatopulmonary syndrome (HPS) with right-to-left (R-L) shunt in cirrhotic patients using the technetium-99m macroaggregated albumin (Tc-99m MAA) lung perfusion scintigraphy (LPS). The authors have found that LPS was more sensitive than contrast-enhanced echocardiography (CEE) for detecting intrapulmonary vascular dilatations (IPVDs) and concluded that Tc-99m MAA LPS can be used complementarily with other diagnostic methods in the assessment of HPS (1). However, we have some concerns regarding this work. The authors appraised the shunt fraction (SF) by using the formula SF=(geometric mean of brain counts)/(geometric mean of brain counts+geometric mean of lung counts), without dividing the geometric mean of brain counts by 0.13, although the brain is presumed to receive 13% of the cardiac output (2). Moreover, both LPS and CEE procedures were not described in sufficient detail.

Apart from the patients’ characteristics, the diagnostic accuracy of LPS for identifying IPVDs can be affected by procedures and protocols. It should be noted that quantification with a technique that uses only brain uptake underestimates SF (2). In a recent prospective study, Zhao et al. (2) compared the whole-body uptake and brain uptake techniques for calculating R-L shunt in 69 patients who received Tc-99m MAA in an upright position to maximize the degree of R-L shunt. The study demonstrated that the whole-body uptake technique has a higher diagnostic accuracy than the brain uptake (74% versus 59%, respectively) for detecting IPVDs in HPS (2). Furthermore, to the best of our knowledge, in all studies assessing patients with HPS, the diagnosis of IPVDs was based only on strict numerical values as expressed by the quantitative analysis, and no qualitative assessment of LPS was made. In a retrospective cohort study, 126 patients with a clinical suspicion of intracardiac R-L shunt underwent LPS (3). A visual scan interpretation demonstrated that the absence of brain parenchymal accumulation of Tc-99m MAA in a static image excluded the R-L shunt, and the specificity of a positive result was 100% (3). Quantitative brain imaging is characterized by a very high specificity and is extremely useful in patients with HPS and concomitant pulmonary diseases (30% of cases), severe and very severe HPS according to the arterial blood-gas analysis (PaO_2_ <60 mmHg), and nondiagnostic CEE (approximately 7%) (2,4,5). Alternatively, although CEE is deemed as a sensitive screening test, it lacks specificity, as many cirrhotic patients with positive results on CEE have normal arterial blood-gas analysis and thus, by definition, have no HPS (4). Thus, differences in sensitivity and specificity for CEE and LPS show that these modalities should be combined to get the most of their characteristics for assessing HPS. Additionally, we should bear in mind that both LPS and transthoracic CEE cannot distinguish IPVDs from other anatomical intrapulmonary shunts, such as pulmonary arteriovenous malformations or intracardiac shunts (5). A standardized and optimized Tc-99m MAA LPS protocol, implementing methods of both quantitative and qualitative evaluations and interpretations, is essential for improving the diagnostic accuracy of HPS.

## References

[1] Alipour Z, Armin A, Mohamadi S, Tabib SM, Azizmohammadi Z, Gholamrezanezhad A, Assadi M (2020). Hepatopulmonary Syndrome with Right-to-left Shunt in Cirrhotic Patients Using Macro-Aggregated Albumin Lung Perfusion Scan: Comparison with Contrast Echocardiography and Association with Clinical Data. Mol Imaging Radionucl Ther.

[2] Zhao H, Tsauo J, Zhang XW, Ma HY, Weng NN, Tang GS, Li X (2020). Technetium-99m-labeled macroaggregated albumin lung perfusion scan for diagnosis of hepatopulmonary syndrome: A prospective study comparing brain uptake and whole-body uptake. World J Gastroenterol.

[3] Graves MW, Kiratli PO, Mozley D, Palevsky H, Zukerberg B, Alavi A (2003). Scintigraphic diagnosis of a right to left shunt in end-stage lung disease. Respir Med.

[4] Rodríguez-Roisin R, Krowka MJ, Hervé P, Fallon MB;, ERS Task Force Pulmonary-Hepatic Vascular Disorders (PHD) Scientific Committee (2004). Pulmonary-Hepatic vascular Disorders (PHD). Eur Respir J.

[5] Angeli P, Bernardi M, Villanueva C, Francoz C, Mookerjee RP, Trebicka J, Krag A, Laleman W, Gines P (2018). EASL Clinical Practice Guidelines for the management of patients with decompensated cirrhosis. J Hepatol.

